# Polarized three-photon-pumped laser in a single MOF microcrystal

**DOI:** 10.1038/ncomms11087

**Published:** 2016-03-17

**Authors:** Huajun He, En Ma, Yuanjing Cui, Jiancan Yu, Yu Yang, Tao Song, Chuan-De Wu, Xueyuan Chen, Banglin Chen, Guodong Qian

**Affiliations:** 1 State Key Laboratory of Silicon Materials, Cyrus Tang Center for Sensor Materials and Applications, School of Materials Science and Engineering, Zhejiang University, Hangzhou 310027, China; 2 Key Laboratory of Optoelectronic Materials Chemistry and Physics, Fujian Institute of Research on the Structure of Matter, Chinese Academy of Sciences, Fuzhou, Fujian 350002, China; 3 Department of Chemistry, Zhejiang University, Hangzhou 310027, China; 4 Department of Chemistry, University of Texas at San Antonio, San Antonio, Texas 78249-0698, USA

## Abstract

Higher order multiphoton-pumped polarized lasers have fundamental technological importance. Although they can be used to *in vivo* imaging, their application has yet to be realized. Here we show the first polarized three-photon-pumped (3PP) microcavity laser in a single host–guest composite metal–organic framework (MOF) crystal, via a controllable *in situ* self-assembly strategy. The highly oriented assembly of dye molecules within the MOF provides an opportunity to achieve 3PP lasing with a low lasing threshold and a very high-quality factor on excitation. Furthermore, the 3PP lasing generated from composite MOF is perfectly polarized. These findings may eventually open up a new route to the exploitation of multiphoton-pumped solid-state laser in single MOF microcrystal (or nanocrystal) for future optoelectronic and biomedical applications.

## Abstract (web summary)

Higher-order multi-photon pumped polarized lasers promise application in future optoelectronic and biomedical applications. Here, the authors demonstrate a polarized three-photon pumped (3PP) microcavity laser in a single host-guest composite MOF crystal via a controllable in situ self-assembly strategy.

## Graphical abstract

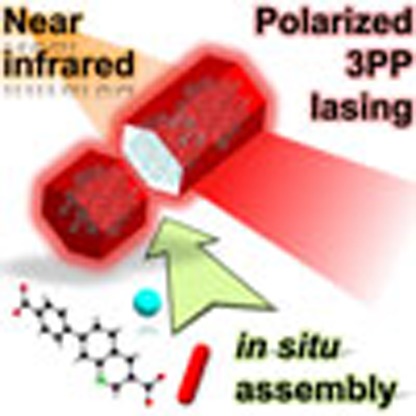

Polarization has been used in various fields, particularly in the field of biophotonics due to its ability to reduce multiple scattering, while to enhance the contrast and to improve tissue imaging resolution^1^,^2^,^3^. Through the measurement of the polarization state of the scattered light, a wealth of structural information of scatters (for example, lesions information in the tissue) can be collected given the fact that the microscopic structure of a scattering media is closely related to changes in the polarization state of the photon during a scattering process^1^,^3^. On the other hand, high-order multiphoton excitation can offer stronger spatial confinement, deeper tissue penetration and less Rayleigh scattering, which are significantly beneficial to the biological imaging^4^,^5^,^6^,^7^,^8^. To make use of the uniqueness of both polarization and high-order multiphoton excitation, the polarized three-photon and/or higher order pumped laser in single solid-state microcrystal is potentially useful for a new kind of biological imaging, so called multiphoton pumped (MPP) polarized emission biological imaging (Supplementary Fig. 1), but has never been realized. In order to produce such a unique laser, the gain medium not only needs to have a high multiphoton absorption (MPA) cross-section and lasing efficiency^4^,^5^, but more importantly needs to be assembled into a suitable microcavity of high concentration and orientation (especially in the case that the absorption transition moment of gain medium is anisotropic) without significant luminescent quenching to enforce the high optical gain and to generate controllable and directional laser. This is really a daunting challenge. In fact, although extensive research endeavours have been pursued to target such a goal, progress has been very slow. The initial effort to diminish the significant quenching effects on the solid state was to homogeneously disperse the gain medium such as the dye molecules with high multiphoton absorption cross-section into its solution^6^,^9^. By employing such a strategy, it still remains extremely difficult to provide with a sufficiently high quenching concentration, which limits the realization of necessary optical gain for compensating the losses. The quenching concentration means that the aggregation-caused quenching (ACQ) gradually becomes dominant when the concentration of gain medium is higher than the quenching concentration. Furthermore, the molecules in the solution are randomly oriented, which would limit their capacities to maximize the optical gain. So far, this dispersed solution methodology can only lead to the amplified spontaneous emission instead of generating three-photon or higher order pumped laser^4^,^5^. Although the luminescent properties of quantum dots are intriguing, they only have generated three-photon-pumped (3PP) random lasing in which the emission direction, position and numbers of mode frequency, and the uniformity of light-emitting region of such lasers are very difficult to control^7^,^10^. Recently, the 3PP lasing from colloidal nanoplatelets in solution has been demonstrated by Li *et al*.^11^; however, no polarization property of the 3PP lasing has been realized. Furthermore, its liquid nature has limited practical applications. To take advantage of the pore confinement of porous materials, zeolites and nanoporous silica have been explored to incorporate dye molecules and semiconducting polymers into the corresponding crystals and thin films to develop solid-state lasing^12^,^13^. However, zeolite/dye composites can only generate single-photon pumped lasing, mainly due to the incompatibility between the inorganic framework and organic guest, leading to the low loading concentration (0.005∼0.0005 M), uneven distribution of dye molecules and poor crystal morphology; while nanoporous silica/semiconducting polymer matrix basically leads to the single-photon pumped polarized amplified spontaneous emission.

Previously, we have used a porous metal–organic framework (MOF) for its pore confinement of a dye molecule bearing moderately high two-photon absorption cross-section, and realized the two-photon pumped lasing from a composite crystal bio-MOF-1⊃DMASM (DMASM=4-[*p*-(dimethylamino)styryl]-1-methylpyridinium) at room temperature^14^. However, the pores (two types of channels along the *c*-axis of about 7.0 and 10.0 Å, respectively) within bio-MOF-1 are still too large to exactly match the dye molecules of DMASM, thus the orientation of the dye molecules inside the pore cavities is not of a high order, particularly when the high concentration of the dye molecules are applied. Such a moderate pore confinement of bio-MOF-1 apparently has limited us to further realize the higher order multiphoton pumped laser in this solid-state crystal. In order to enhance the pore confinement efficiency of a porous MOF crystal, the pore sizes within a porous MOF need to be tuned to match the size of the dye molecule better. But the dilemma is that when the pore sizes of a porous MOF can exactly match the size of the dye molecule, the dye molecules cannot diffuse into the pore channels through the simple post-synthetic exchange process. To overcome this problem, we have developed an *in situ* self-assembly strategy^15^,^16^: the components for building a MOF crystal (metal ion and organic linker) and the organic dye molecule are simultaneously assembled together to form the MOF/dye single crystals. Such a methodology has enabled us to tightly incorporate the dye molecules into the porous MOF crystals, and thus the dye molecules are highly ordered and oriented. We have also managed to immobilize high concentration of the dye molecules into the MOF crystal ZJU-68⊃DMASM ((DMASM)_0.33_H_1.67_[Zn_3_O(CPQC)_3_], CPQC, 7-(4-carboxyphenyl)quinoline-3-carboxylate) (the average pore size of the one-dimensional channel along the *c*-axis is 6.0 Å) with the dye content over 0.4 M. Furthermore, the suitable refraction index and well-faceted MOF composite crystals of certain morphology symmetries can be naturally and efficiently utilized as the laser resonant cavities without any other fabrications. The powerful *in situ* self-assembly strategy, highly efficient pore confinement of ZJU-68 for DMASM dye molecule, and suitable refraction index as well as perfect crystal morphology have enabled us to target the first example of polarized three-photon-pumped laser in single solid-state microcrystal.

## Results

### Synthesis and characterization

**Figure 1:**
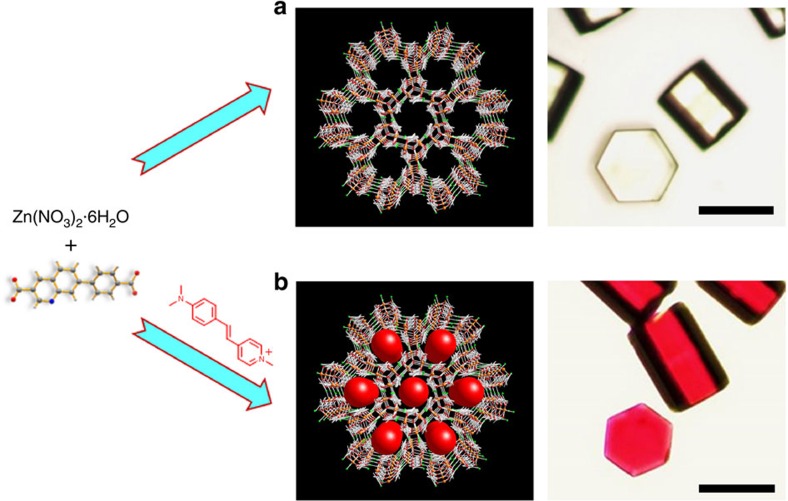
Schematic synthesis of ZJU-68 and ZJU-68⊃DMASM. (**a**) The synthesis and micrograph of a novel metal–organic framework ZJU-68. (**b**) *In situ* synthesis of laser dye incorporated metal–organic framework crystals ZJU-68⊃DMASM. The inclusion of the red dye DMASM molecules leads to the color change from the original colourless ZJU-68 to red ZJU-68⊃DMASM. Scale bar, 50 μm.

**Figure 2:**
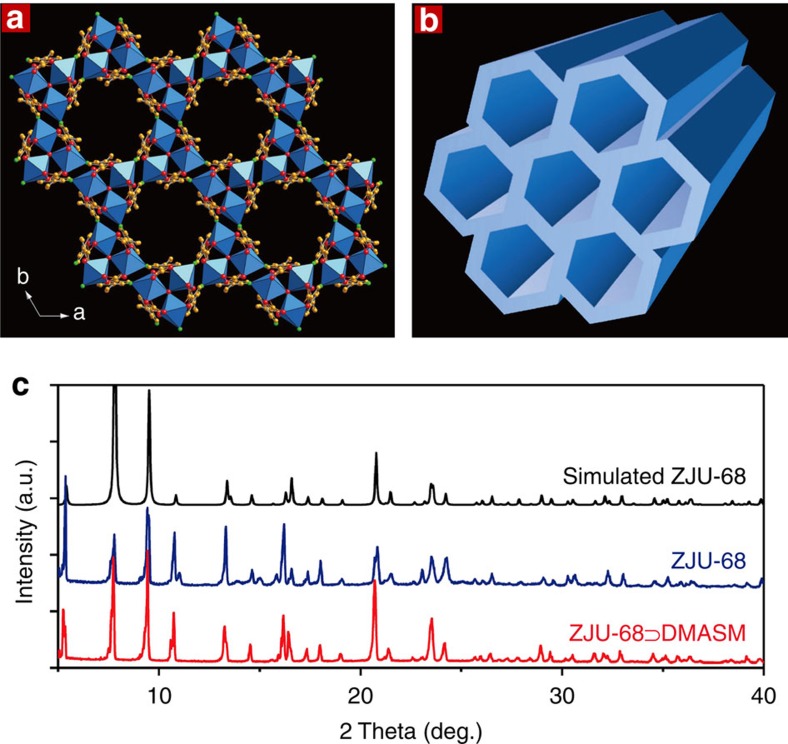
The structure of a novel metal–organic framework crystal ZJU-68. (**a**) Crystal structure of ZJU-68 viewed along the crystallographic c direction (C, orange; N, green; O, red; Zn, blue polyhedra). H atoms and solvent molecules are omitted for clarity. In this structure, nine coordination sites of a trinuclear SBU [Zn_3_O]^4+^ are completely occupied by six carboxylates and three of nitrogen atoms from the quinoline moieties, which may play a crucial role in the stabilization of the resulting MOF, ZJU-68. (**b**) The simplified network structure of ZJU-68, displaying 1D channels along the *c*-axis. Different objects are not drawn to scale. (**c**) PXRD patterns of ZJU-68 and ZJU-68⊃DMASM, which indicate that the ZJU-68⊃DMASM has the identical framework structure with ZJU-68.

Reaction of a new organic linker 7-(4-carboxyphenyl)quinoline-3-carboxylic acid (H_2_CPQC) containing quinolone group and Zn(NO_3_)_2_˙6H_2_O in *N*,*N*-dimethylformamide/acetonitrile/H_2_O/HBF_3_ at 100 ^o^C affords colourless hexagonal prism crystals of H_2_[Zn_3_O(C_17_H_9_NO_4_)_3_]·2.5H_2_O·0.5DMF·MeCN (ZJU-68, Fig. 1a). Single crystal X-ray diffraction studies reveal that ZJU-68 crystallizes in the 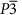 space group (see Supplementary Table 1 for detailed crystallographic data). As shown in Fig. 2a, trinuclear secondary building units (SBUs) of [Zn_3_O]^4+^ are linked by the ligands CPQC^2−^ to form an anionic framework of [Zn_3_O(C_17_H_9_NO_4_)_3_]^2−^. In this structure, nine coordination sites of [Zn_3_O]^4+^ are completely occupied by six carboxylates and three of nitrogen atoms from the quinoline moieties, which are different from most of metal–organic frameworks with [M_3_O]^3*n*–2^ (*n*=3 for M=Cr^3+^, Fe^3+^ and so on or *n*=2 for M=Zn^2+^, Cu^2+^) SBUs in which three sites are occupied by small capping ligands such as water and hydroxide^17^,^18^.

The crystal has one-dimensional (1D) sub-nano channels along the *c*-axis with a hexagonal cross-section (Fig. 2b; Supplementary Fig. 2). The edge of the hexagon is about 3.0 Å. For the synthesis of laser dye functionalized crystals, we tried to introduce linear-shaped laser dye cations DMASM via an ion-exchange process, as described in our previous work^14^, but failed. This is because the DMASM molecule (about 6.3 Å in the width, Supplementary Fig. 2e) is too large to diffuse into the channels of ZJU-68 (ref. 19). We thus developed the *in situ* self-assembly synthetic approach in which the dye DMASM molecules were simultaneously incorporated into framework during the solvothermal synthesis of ZJU-68 by simply adding the dye molecules into the reaction solution (Fig. 1b). The resulting dye DMASM included ZJU-68⊃DMASM has the same hexagonal prism crystal morphology. The inclusion of the red dye DMASM molecules leads to the colour change from the original colourless ZJU-68 to red ZJU-68⊃DMASM. Both single crystal and the powder X-ray diffraction studies (Fig. 2c) confirmed that the ZJU-68⊃DMASM has the identical framework structure with ZJU-68. Furthermore, both ZJU-68 and ZJU-68⊃DMASM demonstrate excellent stability in the air and in the common solvents such as water, ethanol and dimethylformamide (Supplementary Fig. 3). Of course, most of the 1D hexagonal channel spaces have been occupied by DMASM molecules in ZJU-68⊃DMASM. Supplementary Fig. 4 shows the fluorescence micrographs of ZJU-68⊃DMASM, taken by confocal laser scanning microscope. The flat and uniform intensity profiles suggest that the DMASM dyes are homogeneously distributed inside the ZJU-68⊃DMASM composite crystals. The dye contents in this composite can be finely tuned by the addition of different amount of DMASM dyes during the *in situ* self-assembly solvothermal synthesis. Generally speaking, the relatively weak MPA responses require high dye content for MPA lasing measurements^5^,^6^, it is thus necessary to encapsulate as much dye molecules as possible into the pore space of ZJU-68. However, the high dye contents (ingredient mole ratio of *n*_DMASM_/*n*_H_2_CPQC_⩾70%) in the reaction mixtures not only affect the *in situ* self-assembly process (formation of other MOF phases) but also lead to the formation of poor crystalline ZJU-68⊃DMASM. As such, we adjusted the dye concentration in the reaction solution, which produced the optimized ZJU-68⊃DMASM crystals when the ingredient mole ratio of *n*_DMASM_/*n*_H_2_CPQC_ is 35%. Accordingly, per gram of resulting ZJU-68⊃DMASM crystals contain 67.7 mg dye molecules corresponding to the concentration of 0.46 M (the molar amount of dye in per unit volume of solid composite; Supplementary Fig. 5). The optimized ingredient mole ratio (35%) is determined by the measurement of fluorescence quantum yield. Among the ZJU-68⊃DMASM samples with different dye loading concentrations, the ZJU-68⊃DMASM composite crystals with ingredient mole ratio of 35% exhibit the strongest emission at around 635 nm with the highest quantum yield *ϕ* of 24.28±5% on excitation at 450 nm (Supplementary Fig. 6). This is much higher than the quantum yield of 0.45% in dye solutions and of 1.48% solid powder^14^. These results demonstrate that the good confinement of the DMASM molecules within the size-matched channels of ZJU-68 can effectively restrain the intramolecular torsional motion and increase the conformational rigidity of the dye, thus diminishing the ACQ and populating its radiative decay pathway^20^.

### Multiphoton-excited fluorescence in ZJU-68⊃DMASM

**Figure 3:**
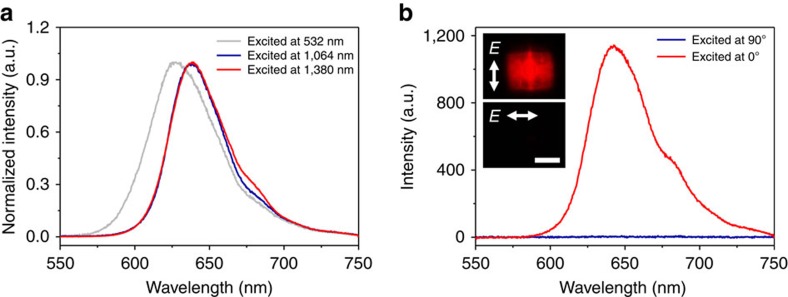
Multiphoton-pumped fluorescence performance of a ZJU-68⊃DMASM single crystal. (**a**) Single-photon-(532 nm), two-photon-(1,064 nm) and three-photon-(1,380 nm) excited emission spectra of ZJU-68⊃DMASM. (**b**) The emission intensity versus pump polarization at two angles *θ*=0° (parallel to the crystal channels) and *θ*=90° (perpendicular to the crystal channels), excited at 1,380 nm. Insets: micrographs of a ZJU-68⊃DMASM single crystal (*R*=36.5 μm) with different pump polarizations excited at 1,380 nm, Scale bar, 50 μm. The high intensity ratio between the two angles indicates the high orientation of dye molecules within the channels of ZJU-68.

Figure 3a compares the single-photon-, two-photon- and three-photon-excited fluorescence spectra of a single ZJU-68⊃DMASM crystal with the dye concentration of 0.46 M under the excitation of a femtosecond laser at different wavelengths. The ZJU-68⊃DMASM shows a strong emission peaked at 627 nm on excitation at 532 nm, whereas the emission peak is red-shifted by 11 to 638 nm when excited at 1,064 nm. The full-width at half-maximum (FWHM) is 53.6 and 42.5 nm, respectively, in the single-photon-, two-photon-excited fluorescence spectra of ZJU-68⊃DMASM. The emission spectrum on excitation at 1,380 nm is basically similar to that excited at 1,064 nm except one additional small peak at around 690 nm attributed to the second harmonic generation response. The red shift of 11 nm under multiphoton excitation can be ascribed to the reabsorption effect^14^. The diffuse reflectance ultraviolet-visible (vis) spectrum of ZJU-68⊃DMASM was shown in Supplementary Fig. 7. There exists overlap between the long wavelength side of the absorption band and the short wavelength side of the fluorescence band (Fig. 3a). Furthermore, all MPP fluorescence bands in Fig. 3a are asymmetric with their left part seeming to be cut off^21^. In addition, the emission peaked at 627 nm from a single ZJU-68⊃DMASM crystal on excitation at 532 nm is blue shifted relative to the spontaneous emission (maximum at 635 nm, see Supplementary Fig. 14) from multiple ZJU-68⊃DMASM crystals, which also suggests the presence of reabsorption effect in ZJU-68⊃DMASM (Supplementary Fig. 8). Fig. 3b shows the fluorescence intensity of the crystal with respect to the pump polarization direction when excited at 1,380 nm. The ZJU-68⊃DMASM exhibits a strong emission when the pump polarization direction is parallel to the crystal channels (along the *c*-axis, denoted as 0°), but hardly emits any light when the pump polarization direction is perpendicular to the excitation direction (90°). Such significant directional fluorescence (dicroic ratio ∼ 365 (ref. 15)) behaviours indicate that the absorption transition moments (approximately along the dye molecule axis^22^) are highly oriented along the crystal channels.

### 3PP lasing in ZJU-68⊃DMASM

**Figure 4:**
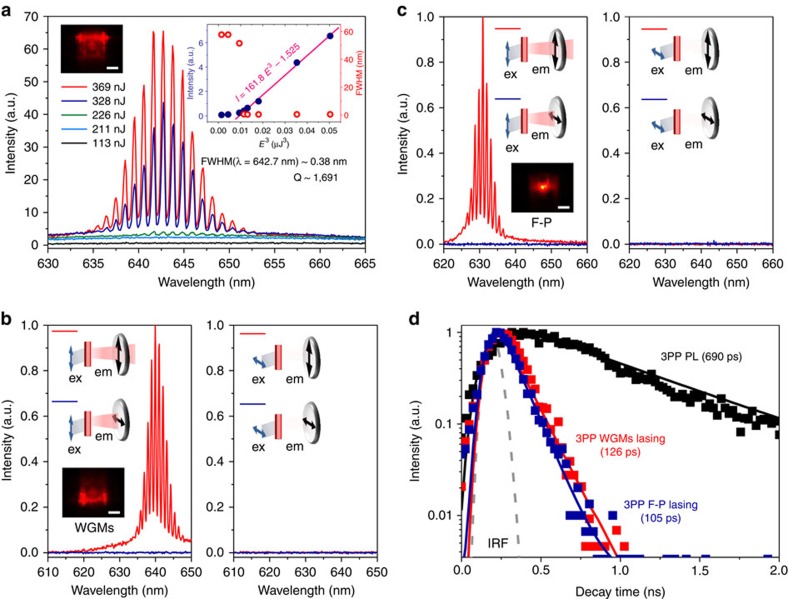
Three-photon-pumped lasing performance of ZJU-68⊃DMASM. (**a**) 1,380 nm pumped emission spectra of ZJU-68⊃DMASM around the lasing threshold. Insets: the micrograph of a ZJU-68⊃DMASM single crystal (*R*=26.2 μm) excited at 1,380 nm (left) and emission intensity and FWHM as a function of pump energy showing the lasing threshold at ∼ 224 nJ (right). At pump energy of 369 nJ, the FWHM of lasing peaked at 642.7 nm is ∼ 0.38 nm, corresponding to a *Q* factor ∼ 1,700. (**b**,**c**) Intensity-dependent emission spectra of 3PP WGMs (**b**, pump energy at 433 nJ) and F–P lasing (**c**, pump energy at 837 nJ) from two isolated crystals (*R*=26.1 and 27.6 μm, respectively) with pump/emission-detected polarization combinations at two angles *θ*=0° (parallel to the crystal channels) and *θ*=90° (perpendicular to the crystal channels), excited at 1,380 nm. Insets: schematic diagrams of the measurement geometry for an individual crystal and micrographs of two kinds of lasing spot patterns due to the F–P (one-spot) and WGMs (two-spot) mechanisms, Scale bar, 20 μm. Both 3PP WGMs and F–P lasing exhibit perfectly polarized emission with DOP >99.9%, which are attributed to the highly oriented assembly of dye molecules within the host–guest composite ZJU-68⊃DMASM microcrystal. (**d**) TRPL decay kinetics measurements of ZJU-68⊃DMASM under photoluminescence (PL), F–P and WGMs lasing excited at 1,380 nm. TRPL, time-resolved photoluminescence

3PP lasing properties were investigated on an isolated single crystal of ZJU-68⊃DMASM with the dye concentration of 0.46 M under a microscope. A femtosecond laser at 1,380 nm was used to pump the crystal at room temperature. This laser beam was directed from a femtosecond optical parametric amplifier (OPA), and then was coupled to the microscope. The emission beam from the crystal was focused and collected with a fibre optic spectrometer. Representative emission spectra near the lasing threshold are shown in Fig. 4a. Under the low-pump energy (*E*) of 113 nJ, the emission spectrum shows a broad peak centred at ∼ 649 nm with a FWHM of 57.6 nm, which corresponds to the spontaneous emission. The pump energy is defined as the laser energy directly received by the MOF crystal (after going through the objective lens and before being incident on the MOF crystal). As the pump energy increases to ⩾230 nJ, a highly progressional emission pattern centred at 642.7 nm appears and grows rapidly with increasing pump energy, while the intensity of the broad spontaneous emission remains almost constant. The visible stimulated emission spectrum centred at 642.7 nm is between one half and one-third of the pumped wavelength of 1,380 nm, which means that the sum energy of two photons at 1,380 nm is not large enough to overcome the bandgap between the ground state (*S*_0_) and excited state (*S*_1_) of ZJU-68⊃DMASM. The stimulated emission of ZJU-68⊃DMASM is therefore induced by the simultaneous absorption of more than two near-infrared photons. To unravel how many photons involved in such a simultaneous absorption process, we further measured the dependence of the stimulated emission intensity on the pump energy. The right inset in Fig. 4a illustrates the pump energy dependence of the fluorescence intensity and FWHM plot as a function of pump energy, giving rise to a linear relationship with cubic pump energy and a low lasing threshold of *E*_th_∼224 nJ as compared with other 3PP stimulated emission^4^,^6^,^7^. The FWHM plot shows a constant value below *E*_th_ and a sudden drop by more than two orders of magnitude when above *E*_th_. The presence of a significant spectral narrowing and a threshold energy coupled with the linearly rapid increase in intensity with cubic pump energy suggest that the 3PP lasing has occurred in the ZJU-68⊃DMASM crystal. The quality factor (*Q*) is given by *Q*=*λ*/δ*λ*, where *λ* and δ*λ* are the peak wavelength and its FWHM, respectively. At pump energy of 369 nJ, the FWHM of lasing peaked at 642.7 nm is∼0.38 nm. This records a high-quality factor *Q* ∼ 1,691 for 3PP lasing, which indicates the high crystal quality supported by our simple chemical approach without etching and coating.

For hexagonal ZJU-68⊃DMASM crystal, the opposing facets can act as the mirrors of a Fabry–Pérot (F–P) cavity, or the six facets can form a whispering gallery modes (WGMs) or other quasi-WGMs cavities^23^ (Supplementary Fig. 9). We observed two kinds of lasing spot pattern on isolated ZJU-68⊃DMASM crystals when excited at 1,380 nm: (1) the strong emission with spatial interference from two-side facets of a hexagonal prism crystal (two-spot pattern)^24^, as shown in the insets of microscopy image in Figs. 4a-4b; the strong emission from a round bright spot at the central facet of the crystal (one-spot pattern)^14^, as shown in the inset of Fig. 4c. Such two lasing patterns can be attributed to the WGMs and F–P feedback mechanisms, respectively, as confirmed later. Fig. 4b,c show the anisotropic study of 3PP WGMs and F–P lasing from two crystals with side lengths of 26.1 and 27.9 μm, respectively. The red emission light from the crystal passed through a polarizer first and then was focused and collected with a fibre optic spectrometer. The schematic diagrams of the measurement geometry for an individual crystal are shown in Fig. 4b,c, where the polarization directions of the pump light and the polarizer are parallel (0°)/perpendicular (90°) to the crystal channels (along *c*-axis). We can see that both 3PP WGMs and F–P lasing with highly structured spectra occur when excited at 0° and emission polarization detected at 0°, while hardly any emission intensity can be detected in all other configurations. The corresponding pump energy is 433 nJ for WGMs lasing and 837 nJ for F–P lasing. It should be noted that the pump energy at almost 3.2 *E*_th_ of 3PP WGMs lasing can realize the 3PP F–P lasing in our experiments, indicating that the WGMs mechanism is more conducive to the realization of 3PP lasing due to the total internal reflection for less loss of light in such size of the crystal. The degree of polarization can be defined as DOP=(*I*_max_−*I*_min_)/(*I*_max_+*I*_min_) in our experiments^25^, and we calculated that both 3PP WGMs and F–P lasing exhibited DOP > 99.9% (limited by the spectral intensity sensitivity of our measurement system) when the excitation polarization is fixed parallel to the crystal channels, indicating a perfectly polarized 3PP lasing operation. Compared with the WGMs lasing, the parallel mirrors in a F–P cavity cannot be utilized as Brewster windows for the polarization selectivity. Therefore, the perfectly polarized 3PP F–P lasing is attributed to the highly oriented assembly of dye molecules within the host–guest composite ZJU-68⊃DMASM microcrystal given the fact that the angle between the absorption transition moment and emission transition moment is close to zero in the dye molecule DMASM^26^. These anisotropic results indicate that ZJU-68⊃DMASM can only be excited at the polarization direction parallel to the crystal channels, and can produce lasing perfectly polarized along the crystal channels, which exhibit a great potential for bioimaging, optical sensing and future optoelectronic integration.

To further confirm the optical-feedback mechanisms for 3PP lasing in these hexagonal ZJU-68⊃DMASM crystals, several single crystals with different side lengths *R* were chosen for the further measurements (Supplementary Fig. 10a and b). For both feedback mechanisms, the spectra of lasing exhibit an increased mode spacing with the decrease of side length of the MOF crystals. For possible resonant modes, the mode spacing Δ*λ*_s_ is defined as^27^


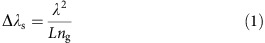


where *λ* is the resonant wavelength, *L* is the cavity path length (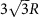 for WGMs and 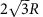 for F–P cavity), and *n*_g_ is the group index of refraction. The measured mode spacing, Δ*λ*_s_, around 635 nm, demonstrates a linear relationship with 1/*R* for each feedback mechanism, which agrees well with Equation (1) (see Supplementary Fig. 10c,d). This result indicates that the lasing with the similar output spot pattern (one-spot or two-spot) can be attributed to the same feedback mechanism. According to the fitting formula, we calculated the ratio of slopes (*S*_one-spot_/*S*_two-spot_) to be 1.47, which is very close to the ratio of the cavity path lengths (*L*_WGMs_/*L*_F–P_=1.5), verifying that the lasing with these two kinds of output pattern (one-spot and two-spot) should be attributed to the F–P cavity and WGMs, respectively. On the basis of Equation (1), we also derived *n*_g_∼3.27 for F–P cavity, and *n*_g_∼3.21 for WGMs at the wavelength of 635 nm. The relatively high group index *n*_g_ value may result from the unusual dispersion relation near the absorption band or the strong exciton–photon coupling in organic materials^28^. Further insight into the 3PP emission performance of the single crystal ZJU-68⊃DMASM arises from time-resolved photoluminescence measurements (Fig. 4d). The pulse durations of the 3PP F–P lasing and WGMs lasing were determined to be 105 and 126 ps, respectively, which are much shorter than the corresponding 3PP fluorescence (below the *E*_th_) decay time of 690 ps. Such temporal narrowing can be ascribed to the depletion in the population inversion of the gain medium with photon-stimulated amplification^6^. The lasing pulse durations from WGMs and F–P are almost in the same order of magnitude, indicating that the optical-feedback mechanism may have little effect on the pulse duration. Subtle differences in our measured decay times of lasing may be ascribed to the proportion of stimulated emission and spontaneous emission in the emitted light, which depends on multiple factors, for example, pump energy, crystal size and crystalline quality^29^,^30^.

## Discussion

In summary, we have achieved an unprecedented solid-state polarized frequency-upconversion lasing in a novel composite single microcrystal ZJU-68⊃DMASM by simultaneous three-photon absorption in the near-infrared region. The tightly confined and highly oriented cationic DMASM dye molecules in anionic ZJU-68 nano-channels through an *in situ* assembly process efficiently increase the loaded concentration, minimize the aggregation and optimize the orientation of dye molecules within the framework, which fulfilled the high-gain lasing with highly polarized excitation response and perfectly polarized emission in a micro-sized laser cavity. Particularly, the 3PP lasing, with a low lasing threshold of ∼ 224 nJ centred at 642.7 nm on excitation at 1,380 nm, has been successfully achieved with a record high-quality factor of ∼ 1,700. Both F–P and WGMs optical-feedback mechanisms have been confirmed to be responsible for 3PP lasing in ZJU-68⊃DMASM microcrystals. Owing to the highly oriented assembly of dye molecules within ZJU-68⊃DMASM, the 3PP WGMs and especially F–P lasing show a perfect emission polarization with DOP > 99.9%. The observed solid-state frequency-upconversion polarized lasing induced by 3PP may find great potentials in practical applications such as photonics, information storage and biomedicine, to name a few. For instance, the wavelength of 1,380 nm belongs to the near-infrared-IIa window (1,300-1,400 nm), which is very promising in biological applications (especially for *in vivo* imaging) because such wavelength region not only can reach deeper penetration depths and minimize the scattering/auto-fluorescence of biological tissues, but also avoid an increased light absorption from water above 1,400 nm (ref. 31). Because the MOF strategy and design can provide us with rich structures of the systematically tuned pore/channel sizes to encapsulate various chromophores with controlled concentration and orientation^32^,^33^,^34^, we anticipate that higher order multiphoton-pumped lasing in solid state can also be realized given that the chromophores (or other nano-sized materials) with great multiphoton absorption properties are well incorporated into the structurally matched MOFs. These findings may eventually open up a new route to the exploitation of multiphoton-pumped solid-state laser in single MOF microcrystal (or nanocrystal) for future optoelectronic and biomedical applications.

## Methods

### Synthesis of ZJU-68⊃DMASM

A mixture of Zn(NO_3_)_2_·6H_2_O (0.34 mmol, 149 mg), H_2_CPQC (0.17 mmol, 50 mg), DMF (10 ml), MeCN (2 ml), H_2_O (0.05 ml), HBF_3_ (0.05 ml) and DMASM iodide (0.03 mmol, 11 mg) were sealed in a 15 ml Teflon-lined stainless-steel bomb at 100 °C for 24 h, which was then slowly cooled to room temperature. After decanting the mother liquor, the fine red hexagonal crystalline product was rinsed three times with fresh DMF (5 ml × 3) and dried in air. The synthesis of the new organic linker H_2_CPQC can be found in Supplementary Fig. 11 and Supplementary Methods.

### Measurements

For MPP, an optical parametric amplifier (TOPAS-F-UV2, Spectra-Physics) pumped by a regeneratively amplified femtosecond Ti:sapphire laser system (800 nm, 1 kHz, pulse energy of 4 mJ, pulse width<120 fs, Spitfire Pro-FIKXP, Spectra-Physics), which was seeded by a femtosecond Ti-sapphire oscillator (80 MHz, pulse width<70 fs, 710-920 nm, Mai Tai XF-1, Spectra-Physics) was used for generating the excitation pulse (1 kHz, 240–2,600 nm, pulse width<120 fs). The incident laser was coupled to the microscope (Ti-U, Nikon), focusing on crystals through an objective lens (CFI TU Plan Epi ELWD 50 × , numerical aperture=0.60, work distance=11.0 mm) with an exposure region of diameter around 30 μm (supplementary Fig. 12). The excited red light was then focused and collected by the fibre optic spectrometer (QE65Pro, Ocean Optics).

The decay curves of multiphoton-pumped emissions were measured by a picosecond lifetime spectrometer (Lifespec-ps, Edinburgh Instruments). For the lifetime measurement of upconverted fluorescence, the pump energy was under the lasing threshold to ensure that no stimulated emission was generated. To measure the decay of the multiphoton-pumped lasing, the pump energy was enhanced over the threshold so that the ultra-strong lasing could be achieved.

Contents of well-dried dye-included ZJU-68⊃DMASM crystals were determined by ^1^H NMR. As shown in Supplementary Figs 5 and 13c, we calibrated and obtained peak area values of peaks that belong to H_2_CPQC and DMASM, respectively. The ratio (*R*_a_) of their peak area values represents the ratio of their contents in the crystal. The dye concentration of the ZJU-68⊃DMASM composite is calculated from *c*=2*R*_a_/*N*_A_*V*, where *V*=2403.91 Å^3^ and *N*_A_=6.02 × 10^23^ mol^−1^ is Avogadro's constant.

## Additional information

**Accession codes**: The X-ray crystallographic coordinates for structure reported in this study has been deposited at the Cambridge Crystallographic Data Centre (CCDC), under deposition number 1046524. These data can be obtained free of charge from the Cambridge Crystallographic Data Centre via www.ccdc.cam.ac.uk/data_request/cif.

**How to cite this article**: He, H. *et al*. Polarized three-photon-pumped laser in a single MOF microcrystal. *Nat. Commun.* 7:11087 doi: 10.1038/ncomms11087 (2016).

## Supplementary Material

Supplementary Information Supplementary Figures 1-14, Supplementary Table 1, Supplementary Methods and Supplementary Reference

Supplementary Data 1 Crystallographic information file for ZJU-68

## References

[b1] Jameson D. M. & Ross J. A. Fluorescence polarization/anisotropy in diagnostics and imaging. Chem. Rev. 110, 2685–2708 (2010). 20232898 10.1021/cr900267p PMC2868933

[b2] Ghosh N. & Vitkin I. A. Tissue polarimetry: concepts, challenges, applications, and outlook. J. Biomed. Opt. 16, 110801 (2011). 22112102 10.1117/1.3652896

[b3] Gurjar R. S. . Imaging human epithelial properties with polarized light-scattering spectroscopy. Nat. Med. 7, 1245–1248 (2001). 11689891 10.1038/nm1101-1245

[b4] He G. S., Tan L. S., Zheng Q. & Prasad P. N. Multiphoton absorbing materials: molecular designs, characterizations, and applications. Chem. Rev. 108, 1245–1330 (2008). 18361528 10.1021/cr050054x

[b5] Guo L. & Wong M. S. Multiphoton excited fluorescent materials for frequency upconversion emission and fluorescent probes. Adv. Mater. 26, 5400–5428 (2014). 24981591 10.1002/adma.201400084

[b6] Zheng Q. D. . Frequency-upconverted stimulated emission by simultaneous five-photon absorption. Nat. Photon. 7, 234–239 (2013).

[b7] Wang Y. . Stimulated emission and lasing from CdSe/CdS/ZnS core-multi-shell quantum dots by simultaneous three-photon absorption. Adv. Mater. 26, 2954–2961 (2014). 24504537 10.1002/adma.201305125

[b8] Hoover E. E. & Squier J. A. Advances in multiphoton microscopy technology. Nat. Photon. 7, 93–101 (2013). 10.1038/nphoton.2012.361 PMC3846297 24307915

[b9] He G. S., Markowicz P. P., Lin T. C. & Prasad P. N. Observation of stimulated emission by direct three-photon excitation. Nature 415, 767–770 (2002). 11845202 10.1038/415767a

[b10] Gomes A. S., Carvalho M. T., Dominguez C. T., de Araujo C. B. & Prasad P. N. Direct three-photon excitation of upconversion random laser emission in a weakly scattering organic colloidal system. Opt. Express 22, 14305–14310 (2014). 24977528 10.1364/OE.22.014305

[b11] Li M. . Ultralow-threshold multiphoton-pumped lasing from colloidal nanoplatelets in solution. Nat. Commun. 6, 8513 (2015). 26419950 10.1038/ncomms9513 PMC4598837

[b12] Vietze U. . Zeolite-dye microlasers. Phys. Rev. Lett. 81, 4628–4631 (1998).

[b13] Martini I. B. . Controlling optical gain in semiconducting polymers with nanoscale chain positioning and alignment. Nat. Nanotechnol. 2, 647–652 (2007). 18654391 10.1038/nnano.2007.294

[b14] Yu J. . Confinement of pyridinium hemicyanine dye within an anionic metal-organic framework for two-photon-pumped lasing. Nat. Commun. 4, 2719 (2013). 24173352 10.1038/ncomms3719 PMC4089137

[b15] Martinez-Martinez V., Garcia R., Gomez-Hortiguela L., Perez-Pariente J. & Lopez-Arbeloa I. Modulating dye aggregation by incorporation into 1D-MgAPO nanochannels. Chemistry 19, 9859–9865 (2013). 23780893 10.1002/chem.201301285

[b16] Martínez-Martínez V. . Highly luminescent and optically switchable hybrid material by one-pot encapsulation of dyes into MgAPO-11 unidirectional nanopores. ACS Photon. 1, 205–211 (2014).

[b17] Mao C. . Anion stripping as a general method to create cationic porous framework with mobile anions. J. Am. Chem. Soc. 136, 7579–7582 (2014). 24836691 10.1021/ja5030723

[b18] Ferey G. . A chromium terephthalate-based solid with unusually large pore volumes and surface area. Science 309, 2040–2042 (2005). 16179475 10.1126/science.1116275

[b19] Zhao C. F., He G. S., Bhawalkar J. D., Park C. K. & Prasad P. N. Newly synthesized dyes and their polymer/glass composites for one-photon and 2-photon pumped solid-state cavity lasing. Chem. Mater. 7, 1979–1983 (1995).

[b20] Cui Y. J. . Dye encapsulated metal-organic framework for warm-white LED with high color-rendering index. Adv. Funct. Mater. 25, 4796–4802 (2015).

[b21] Ren Y. . Synthesis, structures and two-photon pumped up-conversion lasing properties of two new organic salts. J. Mater. Chem. 10, 2025–2030 (2000).

[b22] Weiß Ö. . in Host-Guest-Systems Based on Nanoporous Crystals 544–557Wiley-VCH Verlag GmbH & Co. (2005).

[b23] Wang X. . Whispering-gallery-mode microlaser based on self-assembled organic single-crystalline hexagonal microdisks. Angew. Chem. Int. Ed. 53, 5863–5867 (2014). 10.1002/anie.201310659 24764282

[b24] Braun I. . Hexagonal microlasers based on organic dyes in nanoporous crystals. Appl. Phys. B 70, 335–343 (2000).

[b25] Zhu H. . Lead halide perovskite nanowire lasers with low lasing thresholds and high quality factors. Nat. Mater. 14, 636–642 (2015). 25849532 10.1038/nmat4271

[b26] Gozhyk I. . Polarization properties of solid-state organic lasers. Phys. Rev. A 86, 043817 (2012).

[b27] Choi S., Ton-That C., Phillips M. R. & Aharonovich I. Observation of whispering gallery modes from hexagonal ZnO microdisks using cathodoluminescence spectroscopy. Appl. Phys. Lett. 103, 171102 (2013).

[b28] Takazawa K., Inoue J., Mitsuishi K. & Takamasu T. Fraction of a millimeter propagation of exciton polaritons in photoexcited nanofibers of organic dye. Phys. Rev. Lett. 105, 067401 (2010). 20868009 10.1103/PhysRevLett.105.067401

[b29] Zhang C. . Two-photon pumped lasing in single-crystal organic nanowire exciton polariton resonators. J. Am. Chem. Soc. 133, 7276–7279 (2011). 21517020 10.1021/ja200549v

[b30] Liu X. . Whispering gallery mode lasing from hexagonal shaped layered lead iodide crystals. ACS Nano 9, 687–695 (2015). 25562110 10.1021/nn5061207

[b31] Hong G. S. . Through-skull fluorescence imaging of the brain in a new near-infrared window. Nat. Photon. 8, 723–730 (2014). 10.1038/nphoton.2014.166 PMC5026222 27642366

[b32] Furukawa H., Cordova K. E., O'Keeffe M. & Yaghi O. M. The chemistry and applications of metal-organic frameworks. Science 341, 1230444 (2013). 23990564 10.1126/science.1230444

[b33] Kitagawa S., Kitaura R. & Noro S. Functional porous coordination polymers. Angew. Chem. Int. Ed. 43, 2334–2375 (2004). 10.1002/anie.200300610 15114565

[b34] Chen B., Xiang S. & Qian G. Metal-organic frameworks with functional pores for recognition of small molecules. Acc. Chem. Res. 43, 1115–1124 (2010). 20450174 10.1021/ar100023y

