# Supplementary material for: Polarized three-photon-pumped laser in a single MOF microcrystal
Source: Nat Commun. 2016 Mar 17;7:11087. doi: 10.1038/ncomms11087 (PMC4800435; doi:10.1038/ncomms11087)
Supplement: Supplementary Information — Supplementary Figures 1-14, Supplementary Table 1, Supplementary Methods and Supplementary Reference [file ncomms11087-s1.pdf]

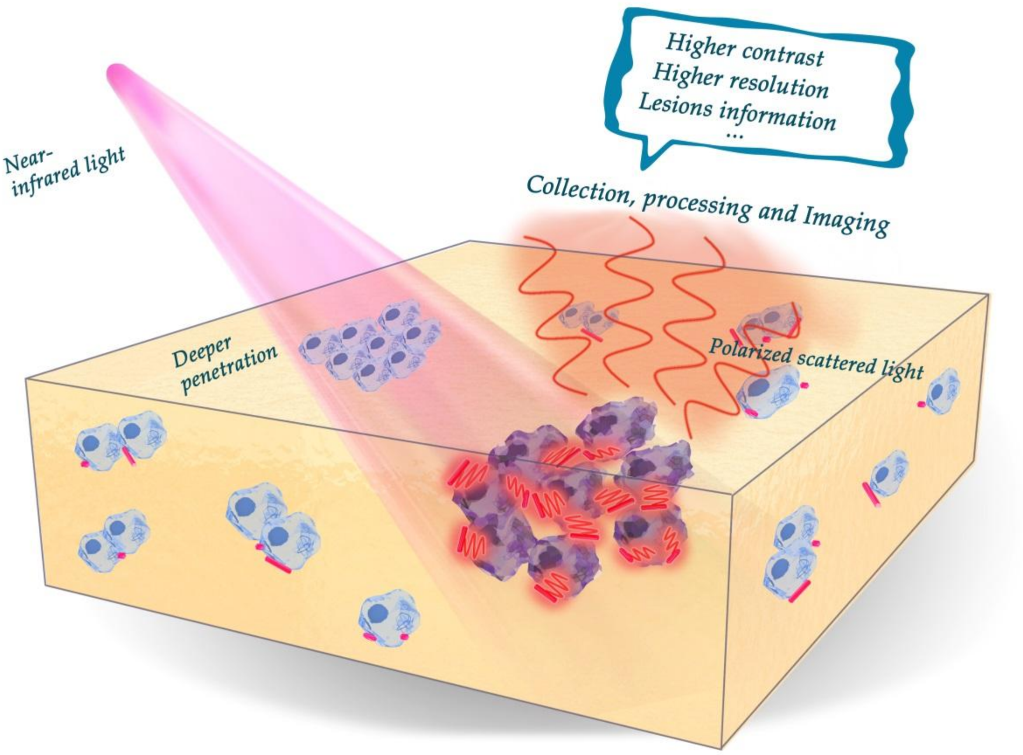

Supplementary Figure 1. The potential application of polarized multi-photon pumped microcavity lasers for biological imaging. The near-infrared excitation can offer stronger spatial confinement, deeper tissue penetration and less Rayleigh scattering; moreover, the collection and imaging of polarized stimulated emission (generated from the microcrystal) passing by the tissue can discriminate against multiply scattered light and report a wealth of structural information, which effectively enhances contrast, improves tissue imaging resolution and even gives lesions information.

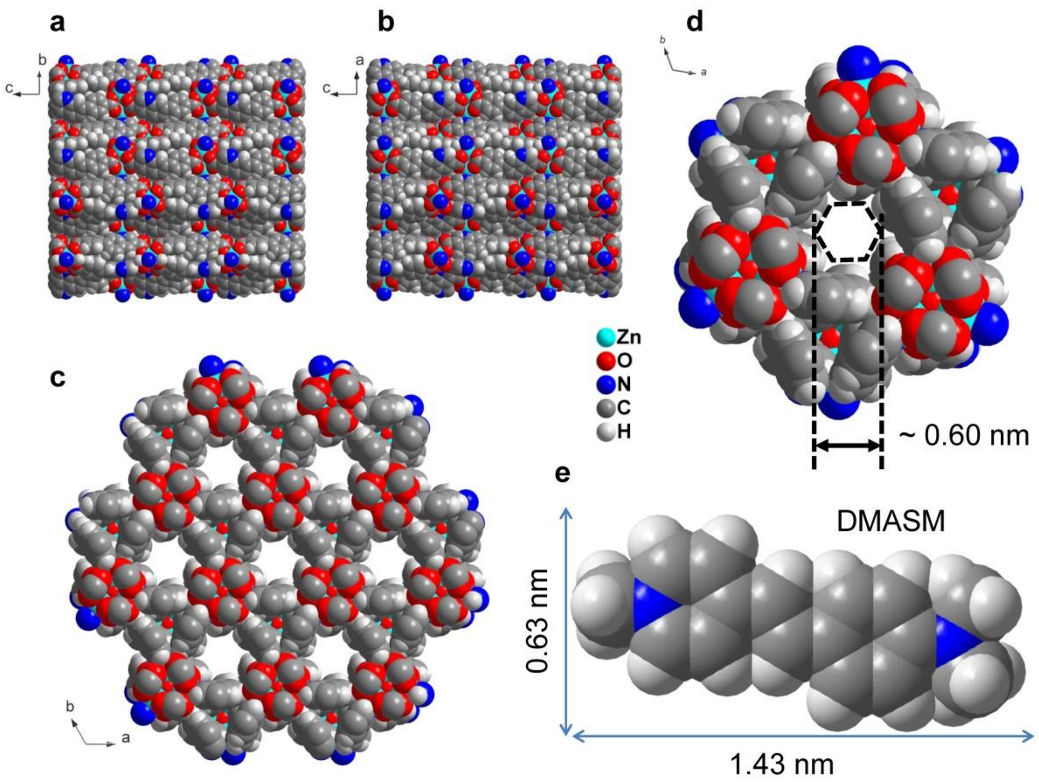

Supplementary Figure 2. (a-c) The space filling model structure of **ZJU-68** viewed along (a) a-axis, (b) b-axis, and (c) c-axis, which shows clearly one-dimensional (1D) channels along c-axis within MOF **ZJU-68**. (d) The 1D sub-nano channels along the *c*-axis have a hexagonal cross-section with the average pore size of about 0.60 nm. (e) The space filling model structure of cationic dye DMASM shows the width of ~ 0.63 nm. Different objects are not drawn to scale. Solvent molecules of crystallization are not shown.

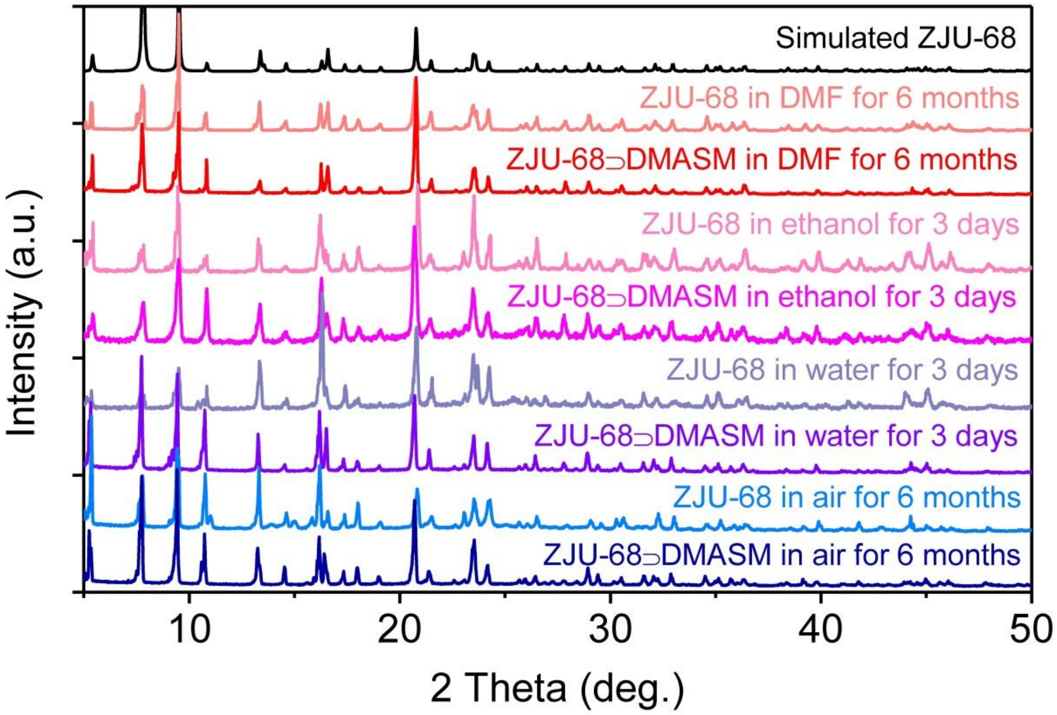

Supplementary Figure 3. PXRD patterns of **ZJU-68** and **ZJU-68⊃DMASM**, which exhibit excellent stability in the air and in the common solvents such as water, ethanol and dimethylformamide.

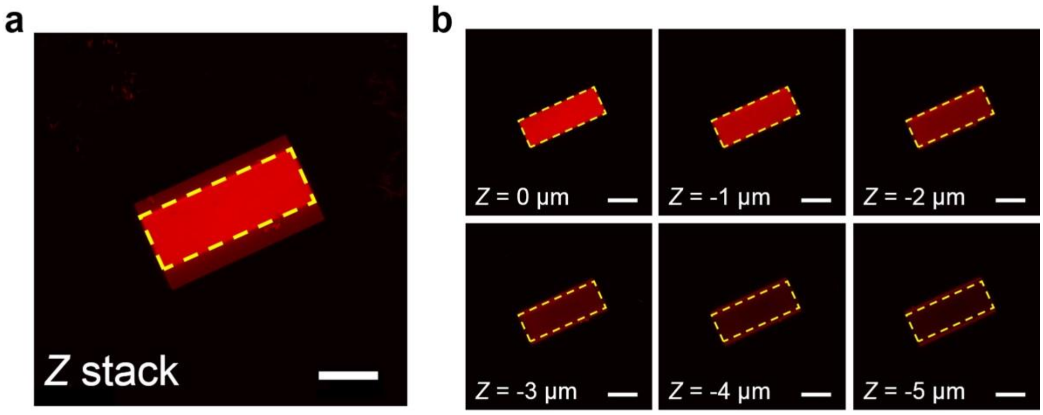

Supplementary Figure 4. The fluorescence micrographs of **ZJU-68⊃DMASM** taken by confocal laser scanning microscope. Scale bar 20 μm. The flat and uniform intensity profiles suggest that the DMASM dyes are homogeneously distributed inside the **ZJU-68⊃DMASM** composite crystals.

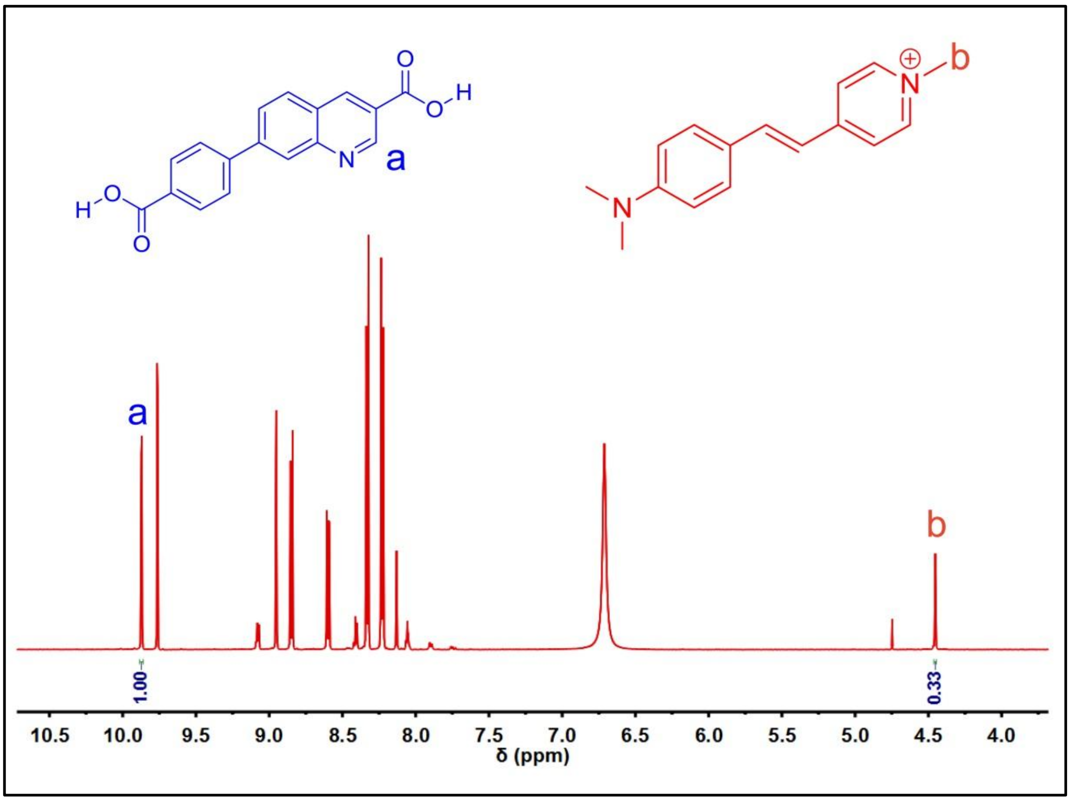

Supplementary Figure 5. The ^{1}H NMR spectrum of **ZJU-68⊃DMASM** used for multiphoton pumped measurements. We calibrated and obtained peak area values of peaks that belong to **H_2CPQC** and DMASM, respectively. The ratio (R_a) of their peak area values represents the ratio of their contents in the crystal. The dye concentration of the **ZJU-68⊃DMASM** composite is calculated to be 6.77 wt% (0.46 M).

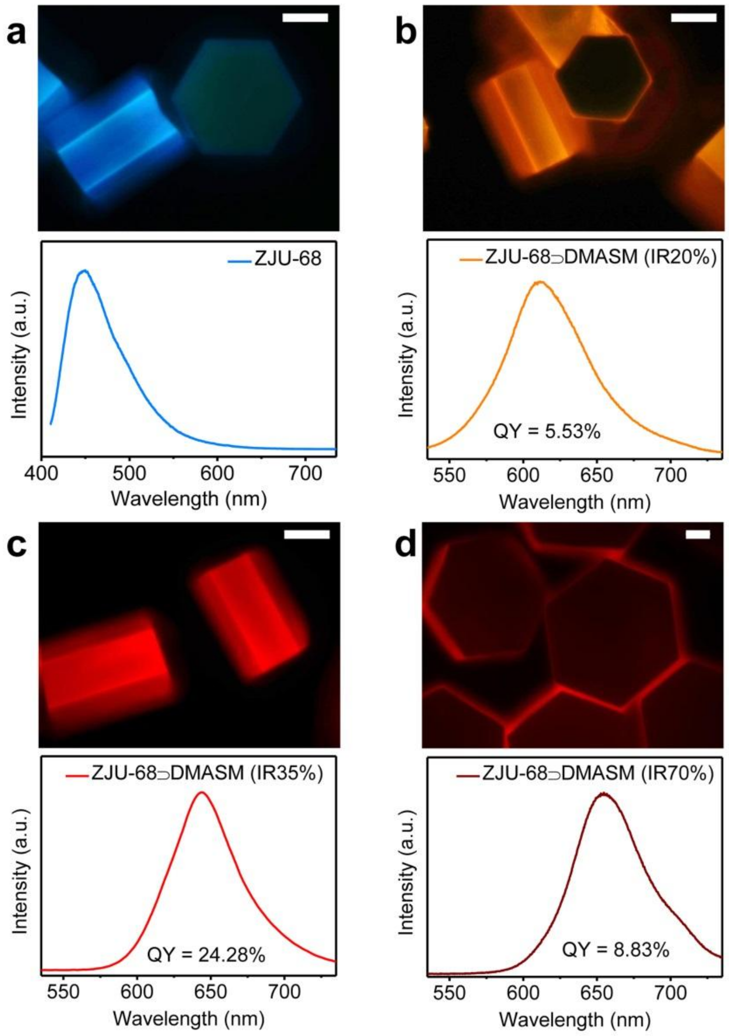

Supplementary Figure 6. Different luminescence colors of **ZJU-68⊃DMASM** with different dye contents. (a) The fluorescence microscopic image and emission spectrum of **ZJU-68** with excitation wavelength of 365 nm. (b-d) The fluorescence microscopic images and emission spectra of **ZJU-68⊃DMASM** with different ingredients ratios (IR, n_{DMASM}/n_{H2CPQC}) of 20% (b), 35% (c) and 70% (d), excited with a 450 nm LED light. It should be noted that the **ZJU-68⊃DMASM** with ingredients ratio of 35% (corresponding to the dye content of 0.46 M) exhibits the highest quantum yield of 24.28% with excitation wavelength of 450 nm. Scale bar, 20 μm.

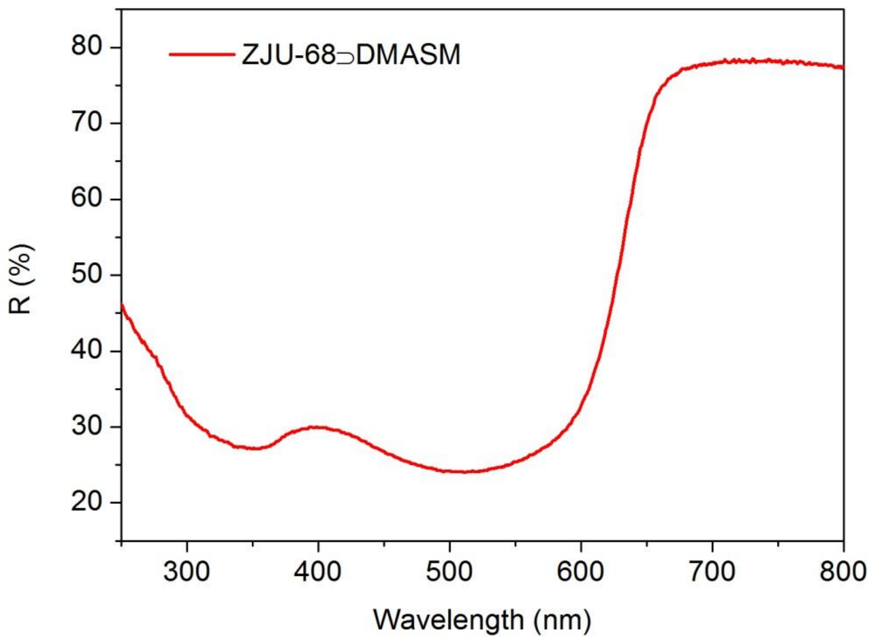

Supplementary Figure 7. Diffuse reflectance UV-vis spectrum of **ZJU-68⊃DMASM**. The spectrum is recorded on a Hitachi U-4100 spectrometer that has equipped with an integral sphere. The absorption band peaks at 350 nm and 512 nm. The former should be attributed to the absorption of MOF **ZJU-68**, while the latter should be ascribed to the absorption of guest dye DMASM.

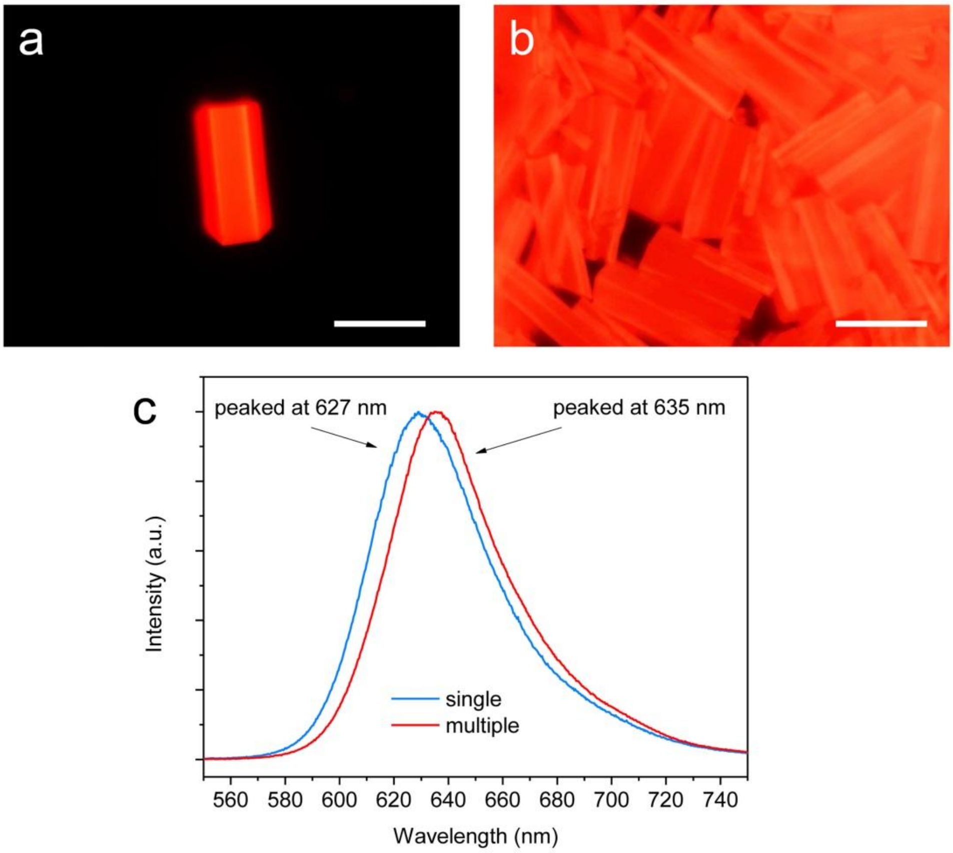

Supplementary Figure 8. Emission spectra difference between **ZJU-68⊃DMASM** single crystal and multiple crystals under excitation at 365 nm. (a-b) The fluorescence microscopic images of single crystal (a) and multiple crystals (b), scale bar, 20 μm. (c) Emission spectra of **ZJU-68⊃DMASM** single crystal and multiple crystals. The red shift of emission maximal peak occurs when exciting multiple crystals suggests that the presence of reabsorption effect in **ZJU-68⊃DMASM** multiple crystals.

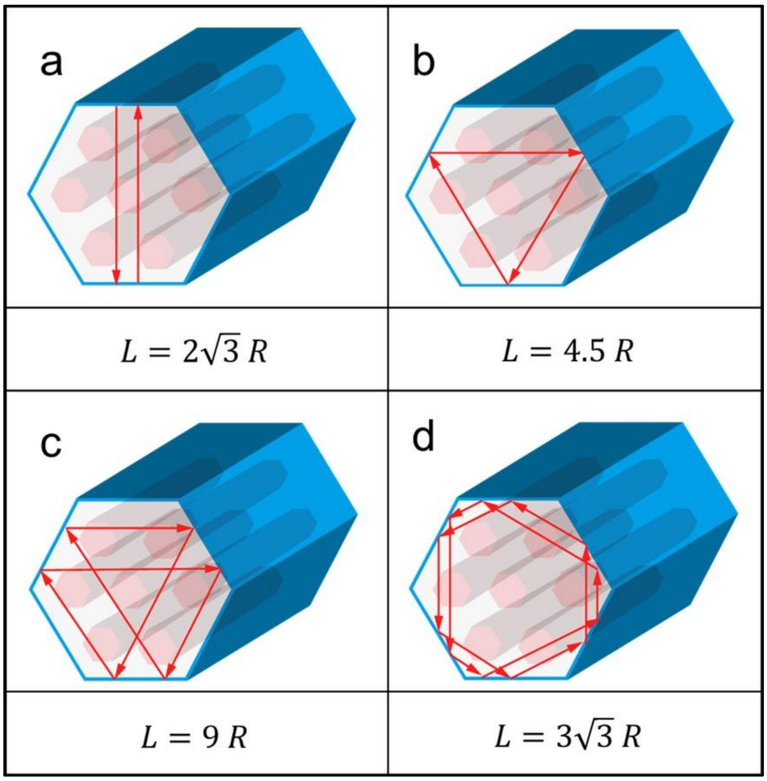

Supplementary Figure 9. Four possible feedback mechanisms with different feedback path lengths (L) in a hexagonal crystal prism[1], (a) F-P cavity, (b-c) quasi-WGMs and (d) WGMs.

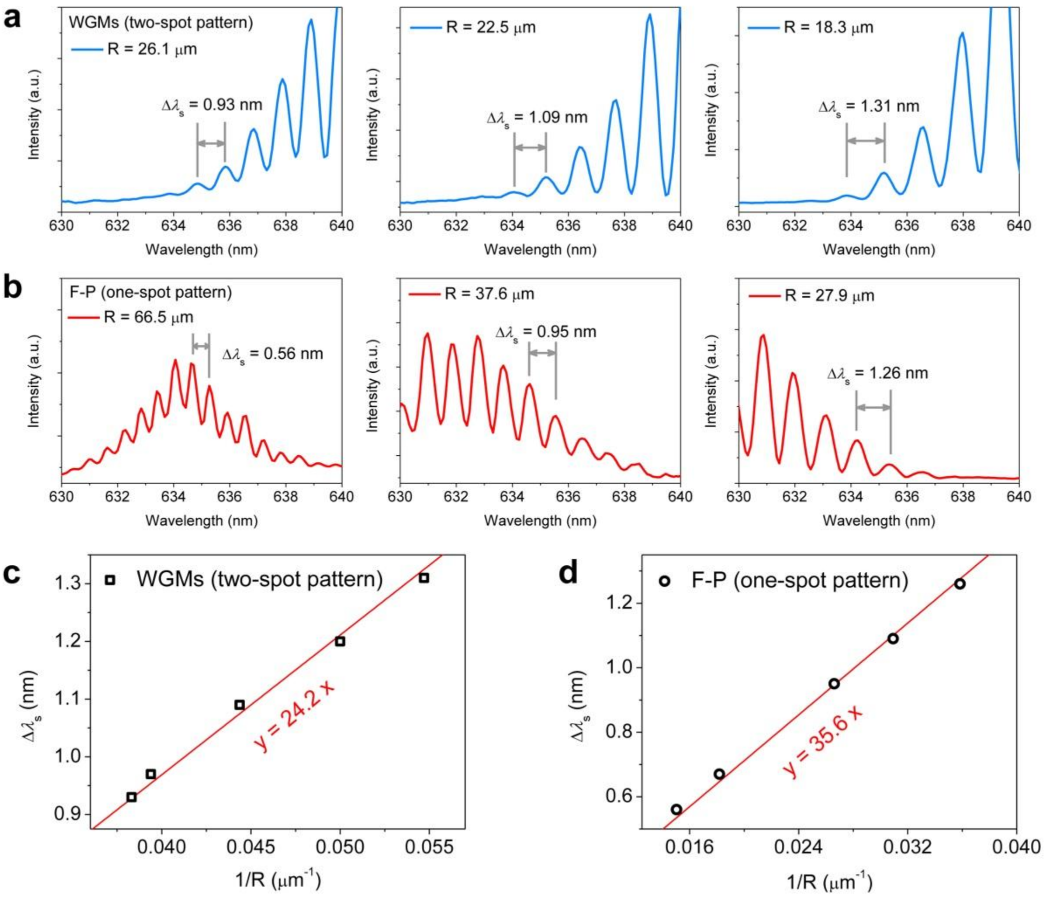

Supplementary Figure 10. The analysis of optical feedback mechanisms towards the 3PP lasing in **ZJU-68⊃DMASM**. (a-b) Emission spectra exhibiting increased mode spacing with the decrease of side length *R* of the MOF crystals from 3PP WGMs lasing (two-spot pattern, a) and 3PP F-P lasing (one-spot pattern, b). (c-d) Mode spacing ($\Delta\lambda_s$, around 635 nm) as a function of the inverse of side length (*R*) towards two 3PP lasing performances (c for two-spot lasing pattern and d for one-spot lasing pattern) with different feedback mechanisms. It should be noted that the ratio of slopes in these two formulas is calculated to be $S_{one\text{-}spot}/S_{two\text{-}spot} = 1.47 \approx 1.5$, consistent with the ratio of the cavity path lengths of the WGMs and F-P cavity mechanisms ($L_{WGMs}/L_{F\text{-}P}$).

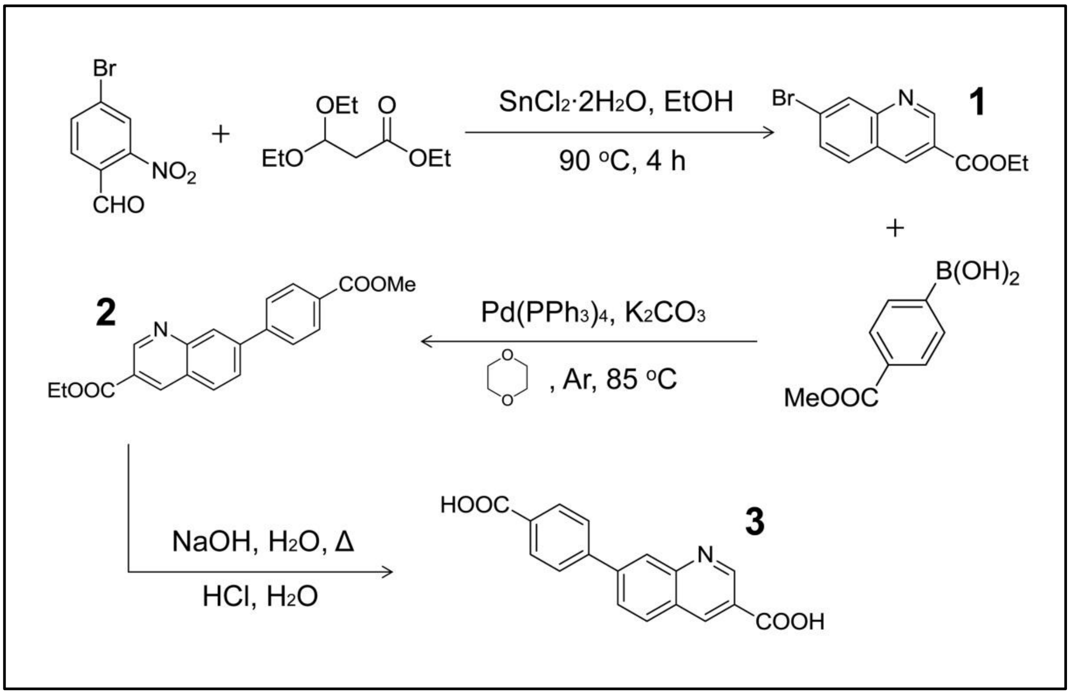

Supplementary Figure 11. Synthetic routes of organic ligand **H_2CPQC**.

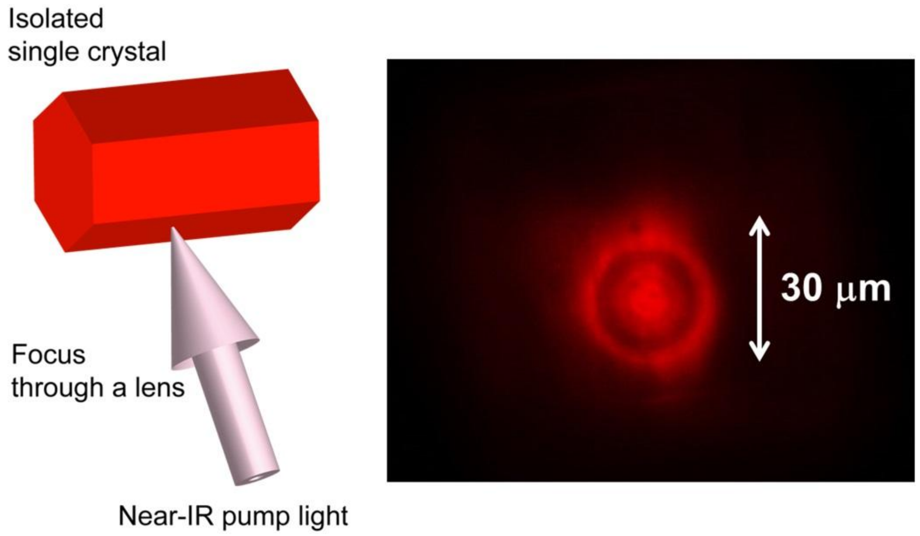

Supplementary Figure 12. The near-IR pump laser focuses on the isolated **ZJU-68⊃DMASM** crystal through the objective lens within the microscope. Because of the focusing, the pump light acting on the crystal is conical like and the diameter of exposure region on the crystal surface is about 30 μm.

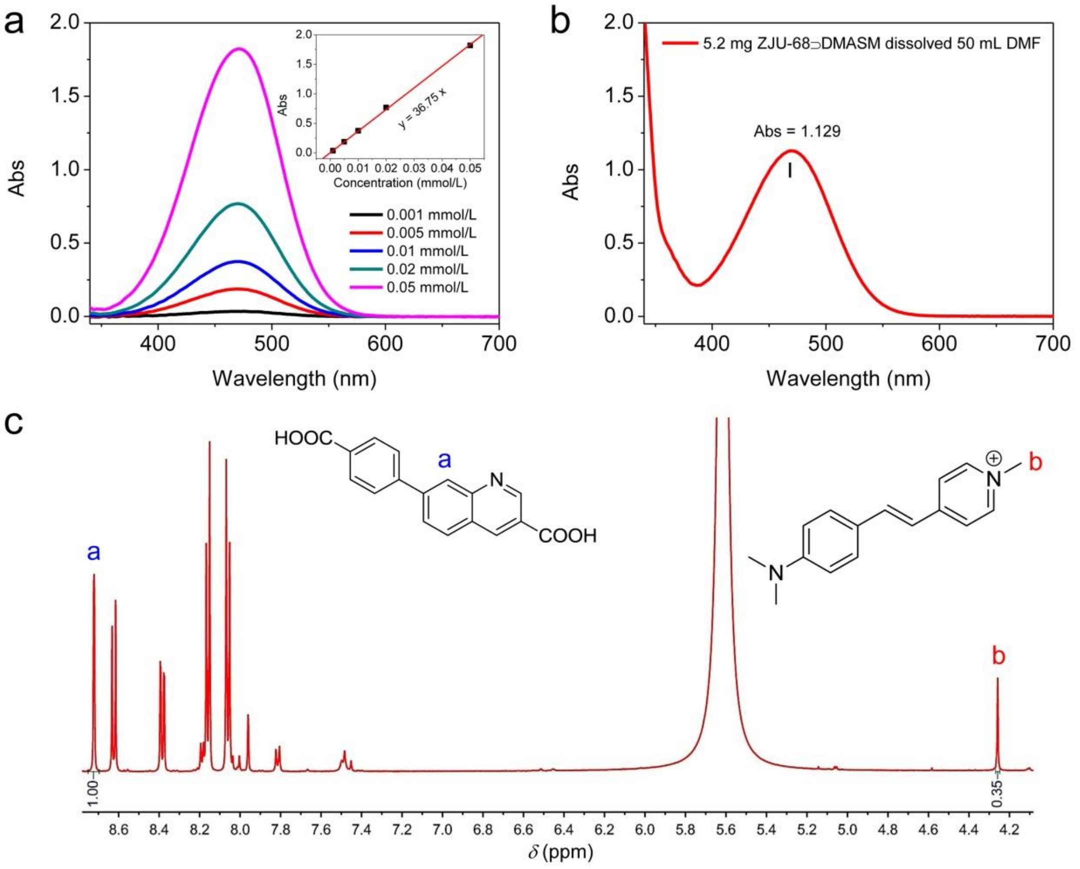

Supplementary Figure 13. Two concentration measurements of the same batch of **ZJU-68⊃DMASM** samples. (a) Absorbance UV-vis spectra of DMASM dye dissolved DMF solution with different dye contents. Inset: dye content dependence of the absorbance peak gives a linear relationship. (b) Absorbance UV-vis spectrum of **ZJU-68⊃DMASM** dissolved DMF solution. Based on (a) and (b), we can calculate the dye content is 7.07 wt% (per gram of **ZJU-68⊃DMASM** crystals contain 70.7 mg dye molecules). (c) The ^{1}H NMR spectrum of **ZJU-68⊃DMASM**. We calibrated and obtained peak area values of peaks that belong to **H_2CPQC** and DMASM, respectively. The dye concentration of the **ZJU-68⊃DMASM** composite is calculated to be 7.15 wt%, which is close to that measured by absorbance UV-vis spectrophotometer.

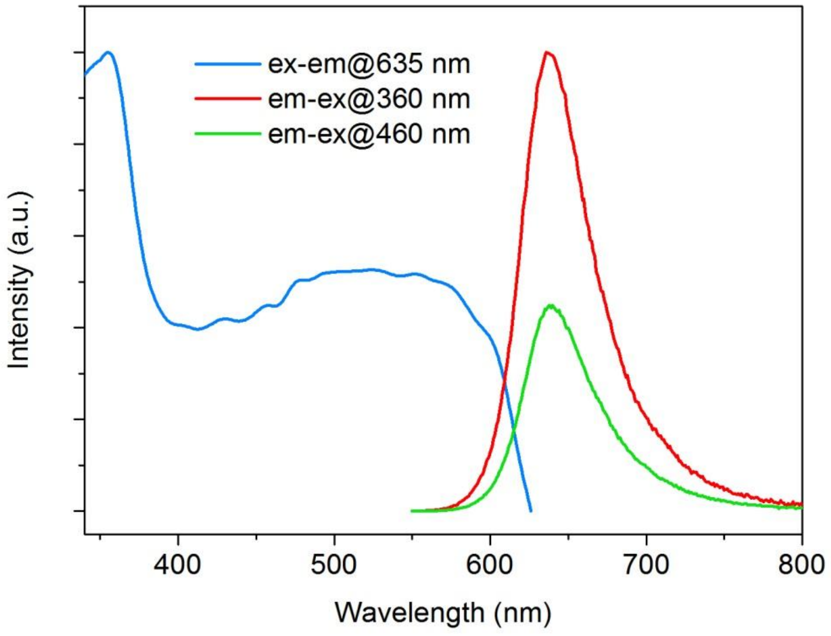

Supplementary Figure 14. The fluorescence spectra of **ZJU-68⊃DMASM** with dye content of 7.1 wt%, taken by the Edinburgh Instrument F900 fluorescence spectrometer.

Supplementary Table 1. Crystallographic data collection and refinement result for **ZJU-68**

**ZJU-68**	
Chemical formula	$C_{51}H_{29}N_3O_{13}Zn_3$
Formula weight	1087.88
Temperature/K	296
Wavelength/Å	0.71073
Crystal system	trigonal
Space group	P-3
a/Å	13.05
b/Å	13.05
c/Å	16.2992
α/°	90
β/°	90
γ/°	120
Z	2
Density(calculated g cm^{-3})	1.503
Absorbance coefficient/nm^{-1}	1.553
Reflections collected	12220
Independent reflections	2941 [Rint = 0.1296]
F(000)	1100
Crystal size/mm^3	0.39×0.31×0.15
Goodness of fit on F^2	1.019
R1,wR2(I>2σ(I))[a]	0.0567, 0.1294
R1,wR2(all date)[a]	0.1279, 0.1448

[a]$R1=\Sigma(|Fo|-|Fc|)/\Sigma|Fo|$;

$wR2=[\Sigma w(|Fo|-|Fc|^2)/\Sigma wFo^2]^{1/2}$.

Supplementary Methods

Materials synthesis

Synthesis of organic ligand H_2CPQC (See Supplementary Figure 11). 4-bromo-2-nitrobenzaldehyde (2.3 g, 10 mmol), ethyl 3,3-diethoxypropanoate (4.85 mL, 25 mmol) and $SnCl_2·2H_2O$ (9.05 g, 40 mmol) were dissolved in EtOH (50 mL). The reaction mixture was stirred and heated to 90 ℃ for 4 h. Upon cooling, the reaction mixture was concentrated and the residue was dissolved in EtOAc and quenched with saturated aq $NaHCO_3$. The resulting emulsion was filtered and rinsed well with EtOAc. The remaining aqueous layer was extracted with EtOAc and the combined organic layers were washed with brine, dried over $MgSO_4$, and concentrated. The yellow solid ethyl 7-bromoquinoline-3-carboxylate was obtained by recrystallization and used for the following reaction. ^1H NMR (500 MHz, $CDCl_3$, δ ppm), 1.47 (t, 3H), 4.48 (q, 2H), 7.74 (d, 1H), 7.81 (d, 1H), 8.38 (s, 1H), 8.83 (s, 1H), 9.46 (s, 1H).

Ethyl 7-bromoquinoline-3-carboxylate (2.8 g, 10 mmol), K_2CO_3 (2.8 g, 20 mmol), and (4-(methoxycarbonyl)phenyl)boronic acid (2.7 g, 15 mmol) were dissolved in dioxane (100 mL). The reaction mixture was stirred under an argon atmosphere for 30 min firstly and then heated to 90 ℃ for 2 days with tetrakis(triphenylphosphine) palladium(0) of 0.35 g (0.3 mmol) added. The resultant precipitate was collected and recrystallized from toluene to obtain off-white solid ethyl 7-(4-(methoxycarbonyl)phenyl)quinoline-3-carboxylate for the following reaction. ^1H NMR (500 MHz, $CDCl_3$, δ ppm), 1.48 (t, 3H), 3.97 (s, 3H), 4.50 (q, 2H), 7.85 (d, 1H), 7.92 (d, 1H), 8.03 (d, 1H), 8.18 (d, 1H), 8.42 (s, 1H), 8.87 (s, 1H), 9.50 (s, 1H).

To the ester ethyl 7-(4-(methoxycarbonyl)phenyl)quinoline-3-carboxylate (6.7 g, 20 mmol) were added NaOH (3.20 g, 80 mmol) in water (50 mL) and dioxane (20 mL). The reaction mixture

was stirred and heated to 90 ℃, and formed a completely clarified solution. Upon cooling, concentrated hydrochloric acid was added dropwise with stirring until the pH of solution reaches about 3. The resultant precipitate was filtered and washed with water thoroughly to afford pale-yellow solid **H_2CPQC**. ^{1}H NMR (500 MHz, DMSO-d_6, δ ppm), 8.06 (d, 1H), 8.10 (d, 1H), 8.13 (d, 1H), 8.32 (d, 1H), 8.44 (s, 1H), 9.03 (s, 1H), 8.36 (s, 1H), 13.3 (s, 1H).

Synthesis of ZJU-68. A mixture of $Zn(NO_3)_2$ $6H_2O$ (0.34 mmol, 149 mg), **H_2CPQC** (0.17 mmol, 50 mg), DMF (10 mL), MeCN (2 mL), H_2O (0.05 mL) and HBF_3 (0.05 ml) was sealed in a 15 mL Teflon-lined stainless-steel bomb at 100 ℃ for 24 h, which was then cooled to room temperature. After decanting the mother liquor, the colorless hexagonal crystalline product was rinsed three times with fresh DMF (5 mL ×3) and dried in air. Elemental analysis, calcd for $H_2[Zn_3O(C_{17}H_9NO_4)_3]\cdot 2.5H_2O\cdot 0.5DMF\cdot MeCN$ ($C_{54.5}H_{40.5}N_{4.5}O_{15.5}Zn_3$, 1202.57) : C, 54.43; H, 3.39; N, 5.24. Found: C, 54.33; H, 3.10; N, 5.12.

***In situ* synthesis of ZJU-68⊃DMASM.** A mixture of DMASM iodide (0.03 mmol, 11 mg), $Zn(NO_3)_2$ $6H_2O$ (0.34 mmol, 149 mg), **H_2CPQC** (0.17 mmol, 50 mg), DMF (10 mL), MeCN (2 mL), H_2O (0.05 mL) and HBF_3 (0.05 mL) was sealed in a 15 mL Teflon-lined stainless-steel bomb at 100 ℃ for 24 h, which was then cooled to room temperature. After decanting the mother liquor, the red hexagonal crystalline product was rinsed four times with fresh DMF (5 mL × 4) and dried in air. Elemental analysis, calcd for $(DMASM)_{0.33}H_{1.67}[Zn_3O(C_{17}H_9NO_4)_3]\cdot 2H_2O\cdot 1.5MeCN$ ($C_{59.28}H_{43.44}N_{5.16}O_{15}Zn_{3,}$ 1264.18): C, 56.32; H, 3.46; N, 5.72. Found: C, 56.29; H, 3.26; N, 5.65.

Measurements

Micrographs and microscopic spectra were taken on an Olympus IX71 inverted fluorescence microscope. Confocal laser scanning images were taken on an Olympus FV1000 laser scanning confocal microscope equipped with an Olympus IX81 inverted microscope.

For multiphoton pumped experiments, an optical parametric amplifier (TOPAS-F-UV2, Spectra-Physics) pumped by a re-generatively amplified femtosecond Ti:sapphire laser system (800 nm, 1kHz, pulse energy of 4 mJ, pulse width < 120 fs, Spitfire Pro-FIKXP, Spectra-Physics), which was seeded by a femtosecond Ti-sapphire oscillator (80 MHz, pulse width < 70 fs, 710-920 nm, Mai Tai XF-1, Spectra-Physics) was used for generating the excitation pulse (1kHz, 240-2,600 nm, pulse width < 120 fs). The incident laser was coupled to the microscope (Ti-U, Nikon), focusing on crystals through an objective lens (CFI TU Plan Epi ELWD 50×, numerical aperture = 0.60, work distance = 11.0mm) with an exposure region of diameter around 30 μm (Supplementary Figure 12). The excited red light was then focused and collected by the fibre optic spectrometer (QE65Pro, Ocean Optics).

The decay curves of multiphoton pumped emissions were measured by a picosecond lifetime spectrometer (Lifespec-ps, Edinburgh Instruments). For the lifetime measurement of upconverted fluorescence, the pump power was under the lasing threshold to ensure that no stimulated emission was generated. To measure the decay of the multiphoton pumped lasing, the pump power was enhanced over the threshold so that the ultra-strong lasing could be achieved.

Quantum yield measurements were performed using the absolute method on a FLS920 from Edinburgh Instruments equipped with a $BaSO_4$-coated integrating sphere, a 450W Xe900 Xenon lamp and a R928P PMT detector. The samples **ZJU-68⊃DMASM** were measured at an excitation

wavelength of 450 nm (Supplementary Figure 6). A 142-mm (inner)-diameter integrating sphere equipped with a cuvette holder and mounts for solid samples and two access ports for the light path was fitted in place of the standard sample holder to collect the excitation and emission light. The absolute quantum yields were calculated by comparing the integral of emission and the absorption of excitation light, with a sensitivity correction for the detector.

Determination of dye contents.

Contents of well-dried dye-included **ZJU-68⊃DMASM** crystals were determined by ^{1}H NMR. As shown in Supplementary Figure 5 and Supplementary Figure 13c, we calibrated and obtained peak area values of peaks that belong to **H_2CPQC** and DMASM, respectively. The ratio (R_a) of their peak area values represents the ratio of their contents in the crystal. The dye concentration of the **ZJU-68⊃DMASM** composite is calculated from $c = 2R_a/N_AV$, where V = 2403.91 Å^3 and N_A = 6.02×10^{23} mol^{-1} is Avogadro's constant.

Supplementary Reference

1 Wang, X. *et al.* Whispering-gallery-mode microlaser based on self-assembled organic single-crystalline hexagonal microdisks. *Angew. Chem. Int. Ed.* **53**, 5863-5867 (2014).
